# Supplementary material for: Targeting fidelity of adenine and cytosine base editors in mouse embryos
Source: Nat Commun. 2018 Nov 15;9:4804. doi: 10.1038/s41467-018-07322-7 (PMC6238002; doi:10.1038/s41467-018-07322-7)
Supplement: Supplementary file 1 — Supplementary Information [file 41467_2018_7322_MOESM1_ESM.pdf]

Supplementary Information

Targeting fidelity of adenine and cytosine base editors in mouse embryos

Lee et al.

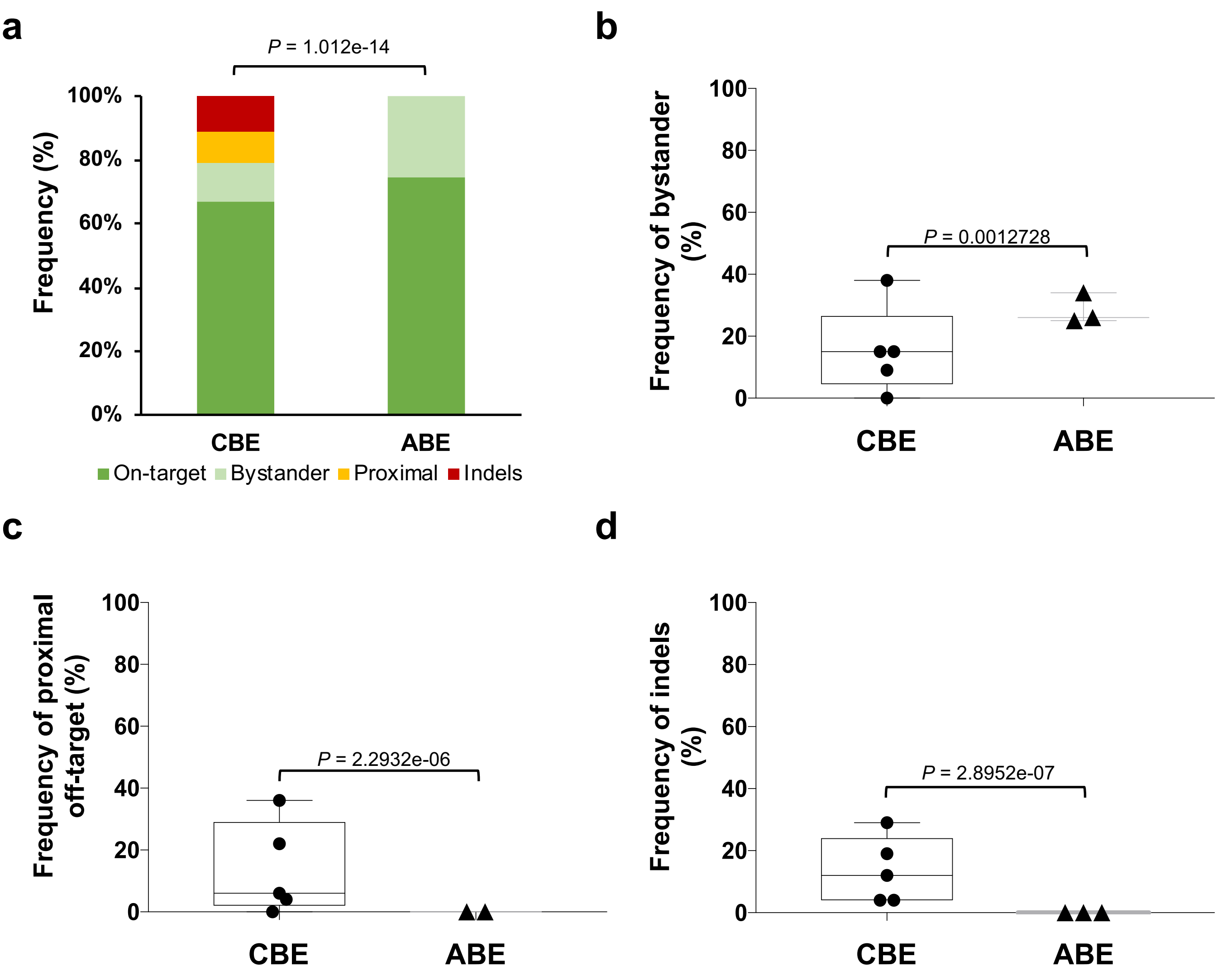

e

Edited alleles		CBE					ABE		
		HF2-BE2	BE3	SaBE3	VQR-BE3	BE4	ABE[a]	ABE[b]	ABE[c]
Bystander	# of alleles	7	8	13	0	4	2	10	56
	# of mutations	8	8	18	0	4	2	10	56
Proximal off-target	# of alleles	17	0	2	5	1	0	0	NA
	# of mutations	39	0	2	8	1	0	0	

Supplementary Fig. 1 Statistical analysis of base editing frequencies. **a** Frequency of alleles carrying different categories of editing outcomes for cytosine base editing (CBE) and adenine base editing (ABE). Note that a single allele can carry more than one mutation. The total number of mutant alleles obtained with CBE and ABE was set as 100% (CBE, 222; ABE, 214). The applied Chi-Squared test is significant (P = 1.012e-14) and the power analysis results in a 83% probability that the test correctly rejects the null hypothesis, using a small effect size and a significance level of 0.05. **b-d** Statistical significance in different categories of off-target mutations. Editing frequencies (Table 1) of each base editor were used the values for comparison of CBE and ABE. Median, middle bar inside the box; IQR, 50% of the data; whiskers, minimum and maximum. Chi-Squared test was applied and each *P-value* was Bonferroni corrected for multiple testing. Bystander editing (b) shows similar frequency for CBE and ABE (P = 0.0012728). In contrast, frequencies of proximal off-targets (P = 2.2932e-06) (c), and indels (P = 2.8952e-07) (d) are highly significant between CBE and ABE. **e** Number of edited alleles carrying bystander and proximal off-target mutations as well as all mutations on edited alleles for each base editor. Some alleles can carry more than one mutation.

a

PMID	Organism	Base editor	Target locus	Mutations
This study	Mouse	VQR-BE3	*Csn2* enhancer C	-10<br>AGAAATGAACAGAGTTCAAAGAAGGCAGGAAAGAGACAAT<br>17x ----------------T-----------------------<br>1x ------T---------T-----------------------<br>1x ---------T------T-----------------------<br>1x -A----T---------T-----------------------<br>1x -T----A---------------------------------<br>1x -T-------T------T-----------------------<br>1x __________________________--------------

b

PMID	Organism	Base editor	Target locus	Mutations
This study	Mouse	BE4	*Csn2* enhancer E	-50<br>TCTCCTT...CAGCCCTTCCTTGTTCACACCCTTTGGGTGACT<br>8x --------TT-----------------------<br>6x --------T------------------------<br>3x ---------T-----------------------<br>2x --------TT-----T-----------------<br>1x -T-----...--------TT-----------------------<br>1x --------TT--------__-------------<br>1x --------TT-------__-----------__-<br>1x --------AT-----------------------<br>1x ---------A-----------------------<br>1x -----------___________-----------<br>1x -------C----_______--------------<br>1x ------------_______--T-----------

c

PMID	Organism	Base editor	Target locus	Mutations
28585179	Mouse	HF2-BE2	*Tyr* (sgRNA-1)	GGTGATGGGAGTCCCTGCGGCCAGCTTTCAGGCAGAGGTTCCTGCC<br>2x --------------------TT------------------------<br>2x ---------------------T------------------------<br>1x --------------------T-------------------------<br>1x --------A-------------------------------------<br>1x ----------------------------T-----------------<br>1x --------C-------------------------------------
			Tyr (sgRNA-2)	C...CTTGTTATTGTGGGAACAAGAAATTCGAGAACTAACTGGGGATGAG<br>12x -------------------------T--------------------<br>5x -------------------------A--------------------<br>2x -----------AAA--------------------------------<br>2x -------------A-----------G--------------------<br>2x -------------------------G--------------------<br>2x --------------------------A-------------------<br>1x G----------AAA-----------G--------------------<br>1x A-----------TA--------------------------------<br>1x T------------A-----------T--------------------<br>1x -----------AAT--------------------------------<br>1x ------------AA-----------T-----------------A--<br>1x ------------AA-----A--------------------------<br>1x -------------A-----A--------------------------<br>1x -------------------------T--A-----------------<br>1x -------------A--------------------------------<br>1x -------------A-----------T--------------------<br>1x -------------T--------------------------------<br>1x T...T----------T-A--T--------G-----------------T--<br>1x -----_____________________--------------------<br>1x -----_____________________--T-G---------------

d

PMID	Organism	Base editor	Target locus	Mutations
28244995	Mouse	BE3	*Dmd*	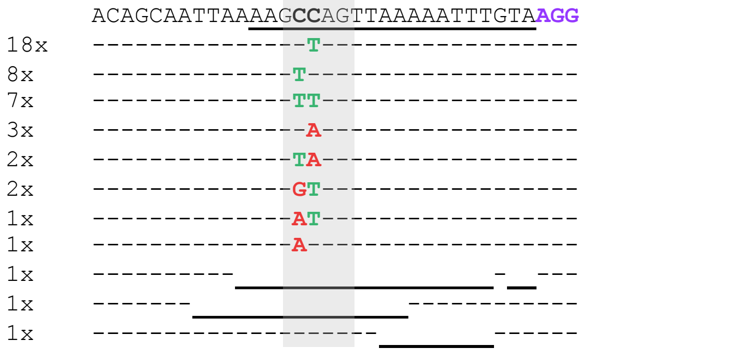
			Tyr	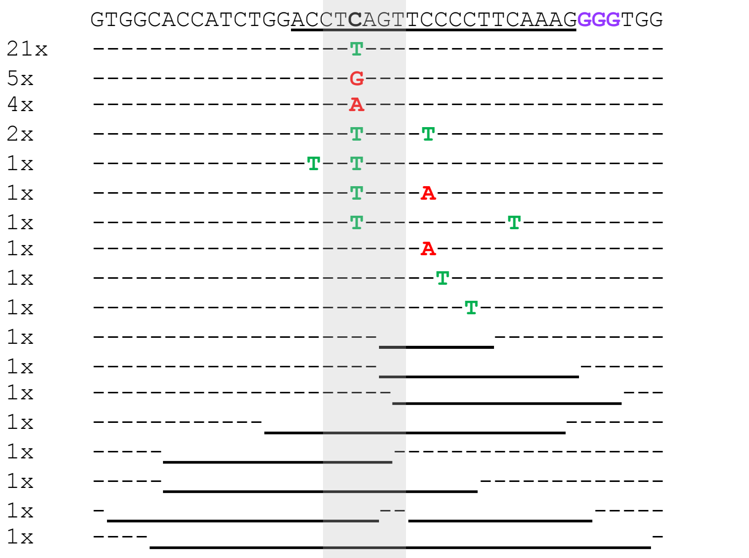

e

PMID	Organism	Base editor	Target locus	Mutations
29904106	Mouse	SaBE3	*Tyr*	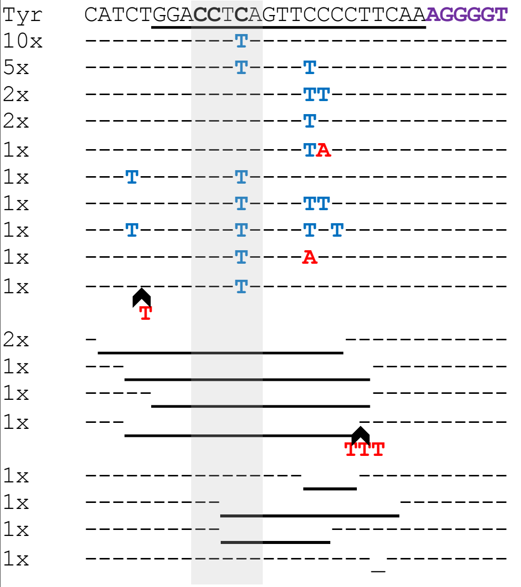

Supplementary Fig. 2 Targeting six loci in the mouse genome with CBEs. **a-e** Alignment of sequences from founder mice or edited embryos injected with VQR-BE3 (a), BE4 (b), HF2-BE2 (c), BE3 (d), or SaBE3 (e), and corresponding sgRNAs. The editing window is shown in grey overlay. The sgRNA sequences are underlined. The target nucleotides for base editing are shown in bold black. The PAM site and nucleotide substituted by cytosine base editing are shown in purple and green, respectively. Opposite-strand edits are labelled in blue and inaccurate mutations are in red. Deletions are shown as underlines.

a

PMID	Organism	Base editor	Target locus	Mutations
This study	Mouse	ABE[a]	*Wap*	GAACAGAGCTCTGGCTCCTAAGACACAGGGCCTTCTGGGAAACTCAAGCAGCC<br>3x ----------------------G------------------------------<br>1x ----------------------G-G----------------------------<br>1x -------------------G---------------------------------<br>1x -------------------GG---G----------------------------
			Csn2 enhancer H	GAGAGTTGCTCTTTCTAATTCTATGAAGAATTGAGTTGGAATTTTGATGGGAT<br>2x ----------------------G------------------------------

b

PMID	Organism	Base editor	Target locus	Mutations
29702637	Mouse	ABE[b]	*Tyr*	CAATGCACCTATCGGCCATAACAGAGACTCTTACATGGTTCCTTTCATACCGC<br>10x -------------------GG--------------------------------<br>10x -------------------G---------------------------------<br>6x -------------------GG-G------------------------------<br>4x -----------------G-G---------------------------------<br>4x -----------------G-GG--------------------------------<br>3x -------------------G--G------------------------------<br>1x -----------------G-G--G------------------------------<br>1x -------------------G--G-G----------------------------

c

PMID	Organism	Base editor	Target locus	Mutations
29904106	Mouse	ABE[c]	*Ar* (sgRNA-1)	Ar-1 TTATCTAGCCTCAATGAGCTTGG<br>34x --G---G----------------<br>19x --G--------------------<br>19x ------G----------------<br>1x --G-G-G----------------<br>1x ----A-G----------------<br>1x --G-T-G----------------
			Ar (sgRNA-15)	Ar-15 ATGATCTCTGCCATCATTTCAGG<br>34x ---G-------------------
			Hoxd13	Hoxd13 GTTTCAGAATCGAAGGGTGAAGG<br>40x -----G-----------------<br>10x -------G---------------<br>7x -----G-G---------------<br>1x -------GG--------------

Supplementary Fig. 3 Targeting three genomic loci using ABE. **a-c** Alignment of sequences from founder mice or embryos derived from edited zygotes that had been injected with ABE and corresponding sgRNAs in our study (a), the *Tyr* locus (b), the *Ar* and *Hoxd13* genes (c). The editing window is shown in grey overlay. The sgRNA sequences are underlined. The target nucleotides for base editing are shown in bold black. The PAM site and nucleotide substituted by adenine base editing are shown in purple and green, respectively. Inaccurate mutations are in red.
